# Anti-Hyperuricemic Effects of Astaxanthin by Regulating Xanthine Oxidase, Adenosine Deaminase and Urate Transporters in Rats

**DOI:** 10.3390/md18120610

**Published:** 2020-12-01

**Authors:** Yanzuo Le, Xie Zhou, Jiawen Zheng, Fangmiao Yu, Yunping Tang, Zuisu Yang, Guofang Ding, Yan Chen

**Affiliations:** Zhejiang Provincial Engineering Technology Research Center of Marine Biomedical Products, School of Food and Pharmacy, Zhejiang Ocean University, Zhoushan 316022, China; yanzuole@zjou.edu.cn (Y.L.); zhouxie1995@zjou.edu.cn (X.Z.); jwzheng1996@zjou.edu.cn (J.Z.); fmyu@zjou.edu.cn (F.Y.); tangyunping1985@zjou.edu.cn (Y.T.); yzs@zjou.edu.cn (Z.Y.); dinggf@zjou.edu.cn (G.D.)

**Keywords:** astaxanthin, fructose, hyperuricemia, adenosine deaminase, xanthine oxidase, urate transporters

## Abstract

This study was designed to investigate the effects and underlying mechanisms of Astaxanthin (AST) on high-fructose-induced hyperuricemia (HUA) from the perspectives of the uric acid (UA) synthesis and excretion in rat models. Following six weeks of a 10% fructose diet, the level of serum UA effectively decreased in the AST groups as compared to the model group. The enzymatic activities of xanthine oxidase (XOD) and adenosine deaminase (ADA) were significantly inhibited, and the mRNA expression levels of XOD and ADA significantly decreased after the AST administration. These results suggested that the AST reduced UA synthesis by inhibiting the mRNA expressions and enzyme activities of XOD and ADA, thereby contributing to HUA improvement. On the hand, the relative expressions of the mRNA and protein of kidney reabsorption transport proteins (GLUT9 and URAT1) were significantly down-regulated by AST, while that of the kidney secretion proteins (OAT1, OAT3 and ABCG2) were significantly up-regulated by AST. These results indicated that the AST promoted UA excretion by regulating the urate transport proteins, and thus alleviated HUA. This study suggested that the AST could serve as an effective alternative to traditional medicinal drugs for the prevention of fructose-induced HUA.

## 1. Introduction

Fructose, a naturally occurring monosaccharide present in fruit, honey, and some vegetables, has plenty of use in processed foods [1]. In recent decades, the daily intake of fructose in the human diet has been increased dramatically, whereas the metabolic process of fructose in the human body produces excessive uric acid (UA), which usually leads to hyperuricemia (HUA), obesity, metabolic syndrome and diabetes [2]. Nowadays, HUA has become a prevalent disease with increasing incidence. The prevalence of gout in American adults has reached to 3.9%, whereas the proportion of young people among the HUA patients is still increasing [3]. HUA is caused by the imbalance of the synthesis and excretion of UA, characterized by high UA levels (serum UA level > 420 µmol/L) [4]. The synthesis of UA primarily occurs in liver. Xanthine oxidase (XOD) and adenosine deaminase (ADA) are the key enzymes responsible for the synthesis of UA in purine metabolism [5]. In hepatocytes, the rapid phosphorylation of fructose requires numerous ATP, which causes reduction in the intracellular phosphate (Pi) level, and consequently the stimulation of AMP deaminase (AMPD) [6]. AMPD catalyzes the hydrolysis of AMP to inosine monophosphate (IMP), which is then metabolized to inosine in the presence of ADA. Inosine is further degraded into hypoxanthine through purine nucleoside phosphorylase (PNP). Eventually, XOD, a rate-limiting enzyme in the purine metabolism, oxidizes hypoxanthine to xanthine and then xanthine to uric acid [6,7]. Hence, the enzymatic activities of XOD and ADA can have significant influence on the synthesis and concentration of serum UA [8].

The accumulated UA is partially excreted out of body by blood circulation, while approximately 70% of the UA excretion occurs through the kidney [9]. The excretion of UA involves the processes of renal secretion and reabsorption, which are controlled by urate transporters. The secretion-related transport proteins include organic anion transporter 1 (OAT1) and organic anion transporter 3 (OAT3), which are highly expressed in the renal proximal tubule and contribute to the transportation of urate [10]. Urate is absorbed by OAT1 and OAT3 from the blood through the basement membrane, which then enters to the renal proximal tubule epithelial cells, and releases through the lumen membrane [11]. Afterwards, the urate is excreted into the lumen of the proximal tubule of nephron with the help of the urate excretion transporter ATP binding cassette superfamily G 2 (ABCG2) [12]. The reabsorption of urate is mainly mediated by glucose transporter 9 (GLUT9) and urate transporter 1 (URAT1). GLUT9 is an important urate transporter, whose major function is to reabsorb urate from the proximal tubule and release it into blood through the basement membrane [13]. URAT1 is another important urate-anion exchanger, which is present in the apical membrane of renal proximal tubule epithelial cells and responsible for the transportation of urate in exchange for Cl¯ or organic anions [14]. Therefore, regulating the expression levels of urate transport proteins could affect the UA excretion and consequently the level of serum UA [15].

Currently, the cornerstone in the prevention of HUA involves the exploitation of drugs, which can promote the excretion of UA or block its synthesis [16]. Allopurinol and febuxostat are the well-known XOD inhibitors, being widely used in the clinics [17]. Diuretics, such as benzbromarone and probenecid, decrease the reabsorption of UA or promote its secretion and serve as anti-HUA drugs [18]. Among all the clinically prescribed drugs, the allopurinol accounts for 98% of the anti-HUA drugs. Nevertheless, these clinical drugs generally have some adverse effects as well, such as allergies, kidney toxicity, liver disease, etc. [19,20]. Therefore, researchers have made tremendous efforts to develop safer anti-HUA drugs from natural products. Park et al. found that ethanol extract of *Aster glehni* inhibited XOD activity and its administration decreased the serum UA level in potassium oxonate-induced HUA rats [21]. Wang et al. reported that cichorium administration promoted the secretion of UA by down-regulating the mRNA and thus expression of ABCCG2, causing the facilitated excretion of UA in HUA rat models [22]. Besides, Young-Sil et al. found that *Alpinia oxyphylla* seed extract improved the reabsorption and secretion of UA in kidney by regulating the UA transport proteins (OAT1, OAT3, GLUT9 and URAT1), which led the attenuation of HUA in rat models [23].

Astaxanthin (Ast), a xanthophyll carotenoid, is mainly derived from the marine organisms [24], and has been confirmed to have anti-inflammatory [25], anti-oxidant [26] and anti-tumor properties [27]. However, the potential effect of AST on HUA has not been investigated yet. Therefore, this study aimed to investigate the preventive effects of AST on HUA caused by a high-fructose diet and its underlying mechanism in rat models. In one aspect, the effects of AST on the UA synthesis were studied. The levels of XOD and ADA in the serum and liver of rat models and the expressions of their corresponding mRNA in rats’ liver were measured. In another aspect, the effects of AST on the regulation of urate transport proteins were investigated by measuring the relative expressions of mRNAs and proteins of reabsorption transport proteins (GLUT9, URAT1) and secretion transport proteins (OAT1, OAT3 and ABCG2) in the rats’ kidneys.

## 2. Results and Discussion

### 2.1. Effect of AST on Serum UA Level

**Figure 1:**
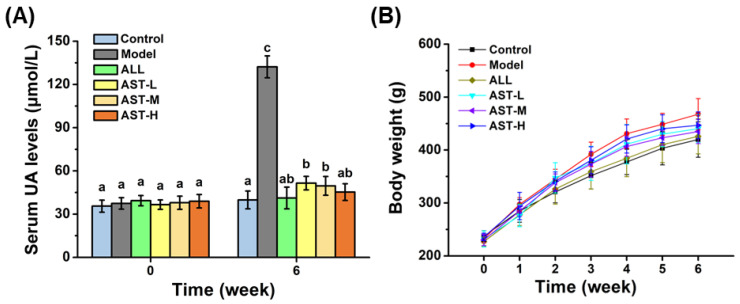
Effects of astaxanthin (AST) on serum uric acid (UA) level (**A**) and body weight (**B**). Values were expressed as mean ± SD (*n* = 8). The different letters indicate significant difference (*p* < 0.05).

The high-fructose diet is a major incentive in the case of HUA [28]. 10% fructose in drinking water is commonly used to induce HUA in rat models [29,30]. In this study, the control group (Control) received normal drinking water, while the other groups were given 10% fructose in drinking water. The other groups were divided into five groups, which included; model group (Model), allopurinol group (ALL), astaxanthin low-dose group (AST-L), astaxanthin middle-dose group (AST-M) and astaxanthin high-dose group (AST-H). The dose range of AST used in this study was 10–40 mg/kg BW/day, which were higher than the proposed amounts in humans. Nevertheless, it should be noted that AST cannot be completely absorbed by the human body because of its poor bioavailability and overdose astaxanthin would be excreted out of body [31]. The levels of serum UA at the start of experimentation and in the sixth week were measured, which are shown in Figure 1. At the start of experimentation, there were no significant differences in the serum UA levels among all the six groups. After six weeks, the level of serum UA in the model group was significantly increased than that of the control group as expected (Figure 1A), indicating the successful establishment of HUA rat models. In comparison with the model group, the levels of serum UA significantly decreased in the ALL and AST groups (Figure 1A). Specifically, the administration of allopurinol lowered the serum UA to the normal level, which was consistent with the previous study [32]. Among all the three AST groups, the AST-H group exhibited the best effect on lowering the serum UA level. The serum UA level of AST-H group showed no significant difference from that of the control group (Figure 1A). The results indicated that the AST group could efficiently decrease the level of serum UA in fructose-induced HUA rat models. The changes in the body weight of rats are shown in Figure 1B. As compared to the control group, the body weights of rats in the model group were significantly increased due to fructose feeding. There were no significant differences in the body weights of rats between the AST groups and the model group, which indicated that the AST had no effect on the body weight (Figure 1B).

### 2.2. Effects of AST on Enzymatic Activities and mRNA Expressions of XOD and ADA in Liver

**Figure 2:**
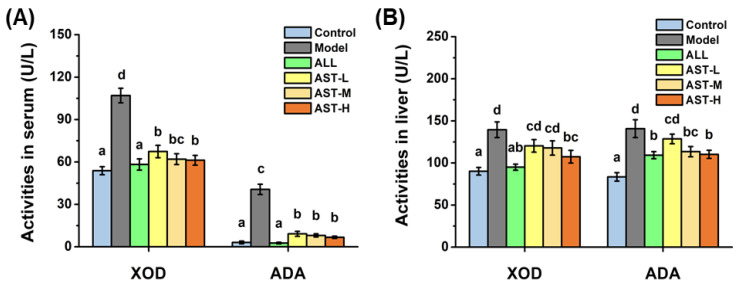
Effects of astaxanthin (AST) on the activities of xanthine oxidase (XOD) and adenosine deaminase (ADA) in serum (**A**) and liver (**B**). Values were expressed as mean ± SD (*n* = 8). The different letters within the “XOD” or “ADA” indicate significant differences in the category (*p* < 0.05).

Blocking the UA synthesis is a key strategy for the treatment of HUA, since the reduction in UA synthesis directly leads to the decrement of serum UA level [33]. XOD and ADA are the key enzymes responsible for the synthesis of UA, which are primarily produced in the liver [34]. In order to demonstrate the inhibitory effects of AST on UA synthesis, the enzymatic activities of XOD and ADA in serum and liver were measured. As shown in Figure 2, the enzymatic activities of both the XOD and ADA in the liver significantly increased in the model group as compared to those in the control group. The high levels of fructose metabolism in liver increased the enzymatic activities of XOD and ADA, which are the pathogenic factors for HUA [35,36]. Whereas, in the ALL group, the allopurinol inhibited the enzymatic activities of XOD and ADA in liver (Figure 2), which was consistent with the previous studies [37,38]. As compared to the model group, the enzymatic activities of XOD and ADA in the AST groups decreased significantly (Figure 2). The trends of the enzymatic activities of XOD and ADA in the serum were remarkably similar to that of the liver (Figure 2). Many studies have reported that the suppression of the enzymatic activities of XOD and ADA can decrease the synthesis of UA to a greater extent [21,39]. He et al. found that the inhibition of the activity of XOD significantly decreased the serum UA level, thereby improving the hyperuricemic condition [40]. Therefore, it is suggested that AST decreases the synthesis of UA by retarding the fructose-induced elevation of XOD and ADA enzymatic activities in both the serum and liver.

**Figure 3:**
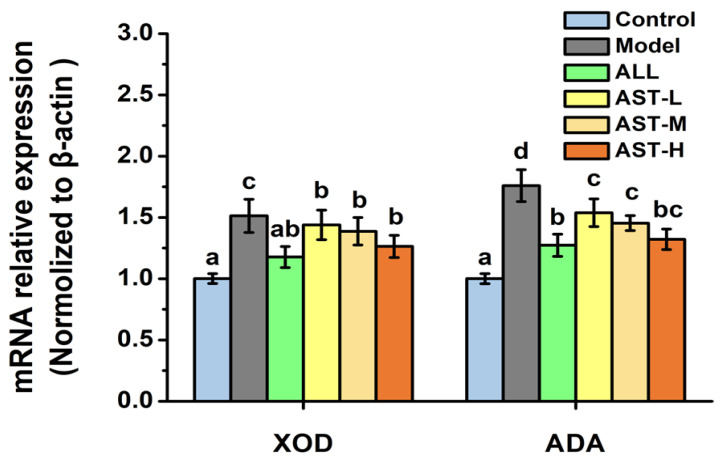
Effects of astaxanthin (AST) on the mRNA relative expressions of xanthine oxidase (XOD) and adenosine deaminase (ADA) in liver. Values were expressed as mean ± SD (*n* = 8). The different letters within the “XOD” or “ADA” indicate significant differences in the category (*p* < 0.05).

**Figure 4:**
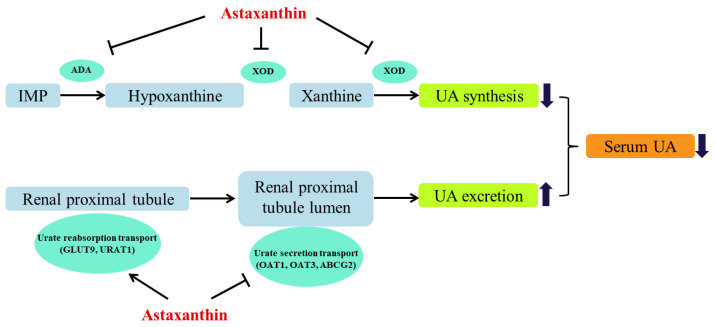
Multitarget role of astaxanthin (AST) in lowering serum uric acid (UA). AST decreased serum UA level by inhibiting xanthine oxidase (XOD) and adenosine deaminase (ADA)-mediated UA synthesis and promoting urate transporters-regulated UA excretion.

As illustrated in Figure 3, the relative expressions of the mRNA of XOD and ADA in the model group increased significantly as compared to the control group. On contrary, the mRNA expressions of XOD and ADA decreased significantly in the AST groups and ALL groups as compared to the model group (Figure 3). The comparison of Figure 2 and Figure 3 suggested that the changes in the enzymatic activities of XOD and ADA were correlated with changes in the relative expressions of the mRNAs of XOD and ADA in liver. The decrease in the enzymatic activities of XOD and ADA was probably associated with the down-regulation of the corresponding mRNA expressions [41]. Therefore, it could be inferred that AST decreased the serum UA level by inhibiting the enzymatic activities and mRNA expressions of XOD and ADA, and thus improving the hyperuricemic condition (Figure 4).

### 2.3. Effects of AST on 24 h Urine Volume and Total UA Excretion

The under-excretion of UA in kidney is another main cause of HUA [42]. In order to determine whether the AST contributed to the excretion of urinary UA, 24 h urine samples were collected and their volume and UA contents were measured. As listed in Table 1, in comparison with the control group, all the other five groups excreted more urine and UA. The increased UA excretion detected in the model group was probably due to the excessive synthesis of UA [43]. Allopurinol inhibits the synthesis of UA by acting on the XOD [44], therefore the 24 h UA excretion of the ALL group had no significant difference from the model group, as listed in Table 1. To be noted, the 24 h UA excretion was significantly elevated in the AST-H group as compared to the model group (Table 1). However, the 24 h UA excretion in the AST-L and the AST-M groups showed no significant differences from that in the model group (Table 1). It has been reported that the increase in the UA excretion could decrease the serum UA level [45]. Therefore, these results suggested that the AST increased UA excretion, which further contributed to lowering the serum UA level and subsequently alleviating the HUA.

### 2.4. Effects of AST on the Levels of Serum Blood Urea Nitrogen (BUN) and Creatinine (Cr)

**Figure 5:**
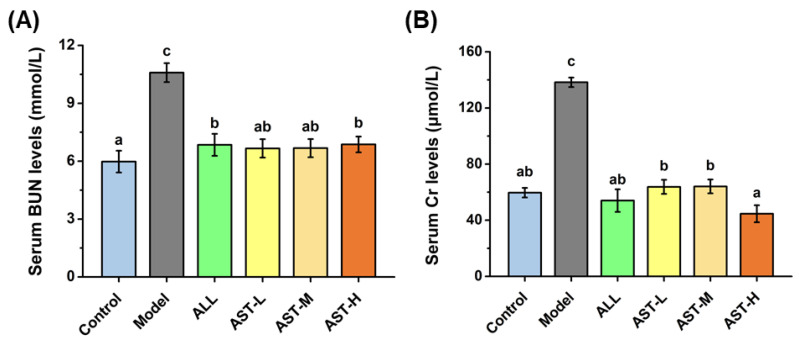
Effects of astaxanthin (AST) on the levels of blood urea nitrogen (BUN) (**A**) and creatinine (Cr) (**B**) in serum. Values were expressed as mean ± SD (*n* = 8). The different letters within the “BUN” or “Cr” indicate significant differences in the category (*p* < 0.05).

The level of serum UA is known to be strongly associated with the excretory function of kidney [9]. The excretory function of kidney consists of three main processes: glomerular filtration, tubular secretion and reabsorption [46]. Normally, the UA is filtered through glomerulus in the kidney. The decrease in the ability of glomerular filtration can decrease the UA excretion and further elevate the serum UA level [47]. In clinical diagnostics, both the BUN and Cr are commonly used indicators for the evaluation of glomerular function [46]. The results of serum BUN and Cr are shown in Figure 5. In the sixth week, the BUN and Cr increased significantly in the model group as compared to the control group. Cr is a small molecular weight protein that is freely filtered by the glomerulus and is not reabsorbed but undergoes only tubular secretion [48]. It is often used to noninvasively estimate the glomerular filtration. Furthermore, previous studies have reported that the BUN level correlates with the reduction of glomerular filtration [49,50,51]. Therefore, the increase in the levels of serum BUN and Cr indicated the impaired capacity of glomerular filtration. After the AST treatment, the levels of BUN and Cr in serum effectively decreased (Figure 5). These results inferred that the AST may maintain the normal function of glomerular filtration in the kidney.

### 2.5. Effects of AST on mRNA and Protein Expressions of Urate Transporters in Kidney

**Figure 6:**
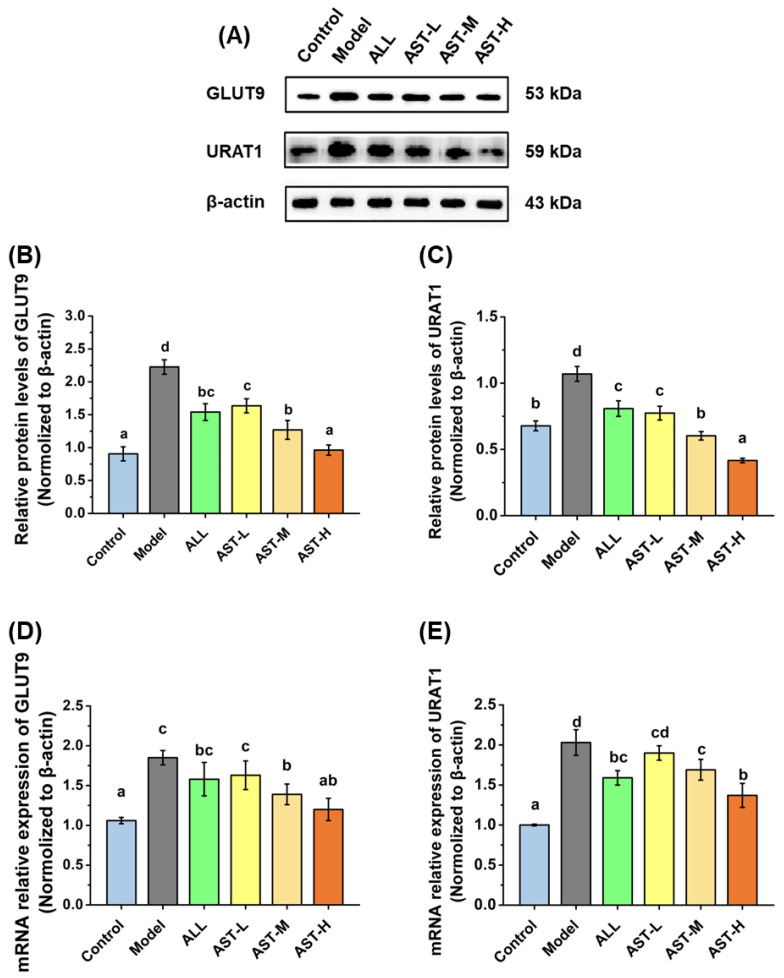
Effects of astaxanthin (AST) on protein relative expressions (**A**–**C**) and mRNA relative expressions (**D**,**E**) of URAT1 and GLUT9 in the kidney. Values were expressed as mean ± SD (*n* = 8). The different letters indicate significant difference (*p* < 0.05).

After the glomerular filtration, UA undergoes the processes of reabsorption and secretion in renal tubules, where almost 90% of the UA is reabsorbed into the blood, while the rest is excreted in urine [52]. The excretion of UA is achieved by the interaction between the reabsorption and secretion of UA in the kidney, and is associated with the various transporter proteins in kidney [15,53]. The major transporter proteins associated with the renal reabsorption of UA are URAT1 and GLUT9. The relative expression levels of the mRNA and protein of URAT1 and GLUT9 were measured and are shown in Figure 6. In the model group, the relative expression levels of the mRNA and protein of URAT1 and GLUT9 in kidney were significantly elevated as compared to that of the control group (Figure 6). This result suggested that the high-fructose diet could enhance the reabsorption of UA in kidney, which is supported by previous findings [30,54]. In contrast to the model group, the AST administration significantly down-regulated the mRNA and protein expression levels of URAT1 and GLUT9 (Figure 6). The down-regulation of the protein expressions of URAT1 and GLUT9 was probably ascribed to the decreased expressions of the mRNA of the corresponding genes. As reported previously, the GLUT9 and URAT1 are responsible for the reabsorption of over 90% of UA into the cells in the proximal tubules of kidney [14,55]. The inhibition of GLUT9 and URAT1 has been reported to accelerate the excretion of UA by reducing the reabsorption of UA in HUA rats [53]. Therefore, the AST may decrease the reabsorption of UA by down-regulating the expression of mRNA and protein levels of GLUT9 and URAT1 and thus decreasing the UA level in serum.

**Figure 7:**
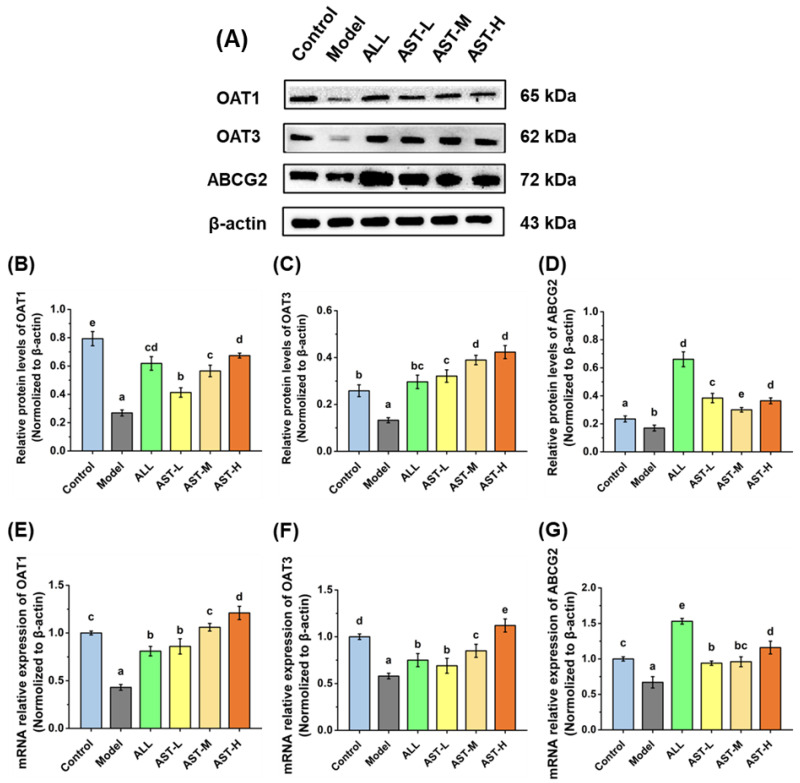
Effects of astaxanthin (AST) on protein relative expressions (**A**–**D**) and mRNA relative expressions (**E**–**G**) of OAT1, OAT3 and ABCG2 in the kidney. Values were expressed as mean ± SD (*n* = 8). The different letters indicate significant difference (*p* < 0.05).

OAT1 and OAT3 are mainly involved in the secretion of UA into the renal proximal tubules, while their specific function is to secrete UA into the epithelial cells through the basolateral membrane [10]. In the model group, the relative expression of the mRNA and protein of OAT1 and OAT3 decreased significantly (Figure 7). Habu et al. reported that the activity of organic anion at the basolateral membrane decreased whereas the proteins of OAT1 and OAT3 were down-regulated in 5% oxonic acid-induced HUA rats [56]. As compared to the model group, both AST and ALL significantly up-regulated the mRNA and protein relative expressions of OAT1 and OAT3 (Figure 7). Among these groups, the AST-H group had the greatest capacity to up-regulate the secretion proteins than the other groups. Wang et al. found that the improvement of UA excretion by the up-regulation of OAT1 and OAT3 expression effectively lowered the concentration of serum UA [57]. ABCG2 is known as a high-capacity urate secretion transporter protein, and its activity is closely associated with the serum UA level due to the genetic polymorphisms [57]. In the model group, the relative expressions of the mRNA and protein of ABCG2 decreased significantly (Figure 7). The allopurinol administration significantly increased relative expression of the mRNA and protein of ABCG2 (Figure 7). Similarly, the relative expressions of the mRNA and protein of ABCG2 were significantly up-regulated in the AST groups. Matsuo et al. studied various ABCG2 transporter mutants in comparison with the wild-type ABCG2 and found that the decrease in the activity of urate transporter protein was proportional to the decrease in the expression of ABCG2 protein [58]. The dysfunction of ABCG2 remarkably increases the risk of HUA and even gout, particularly in young people [59]. The up-regulation of ABCG2 expression in kidney and ileum benefited to the elimination of UA from the body in the HUA rats [12]. In this study, the AST administration significantly increased the mRNA and protein expressions of secretion proteins (OAT1, OAT3 and ABCG2), indicating the facilitated secretion of UA in the high fructose-induced rat models. In conclusion, the AST administration promotes the UA excretion by inhibiting the UA reabsorption and enhancing the UA secretion, which results in lowering the serum UA level and, consequently, improving hyperuricemic condition (Figure 4).

## 3. Materials and Methods

### 3.1. Materials

Astaxanthin was offered by Jingzhou Natural Astaxanthin Inc. (Hubei, China). Allopurinol was purchased from Aladdin (Shanghai, China). Fructose (99%) was offered by Shanghai Macklin Biochemical Technology Co., Ltd. (Shanghai, China). The rabbit anti-rat polyclonal primary antibodies for URAT1, GLUT9, OAT1, OAT3, ABCG2, β-actin and horseradish peroxidase-conjugated goat anti-rabbit IgG secondary antibody were provided by Beijing Bioss Biological Technology Co., Ltd. (Beijing, China). The kits for UA, XOD, ADA, Cr and BUN were purchased from Jiancheng Bioengineering Institute (Nanjing, China). HiScript III RT SuperMix for qPCR (+gDNA wiper) was purchased from Nanjing vazyme Biological Technology Co., Ltd. (Nanjing, China). TRIzol reagent was purchased from Shanghai Thermo Fisher (Shanghai, China). All reagents used in this study were of analytical grade.

### 3.2. Animals, Hyperuricemia Induction and Experimental Design

The experiments on rats were conducted according to the Animal Research Committee of Zhejiang Ocean University, (Laboratory Animal Certificate No. SCXK ZHE 2019-0031). The experiments were conducted on male Sprague-Dawley (SD) rats with body weight around 230 ± 10 g, purchased from Experiment Animal Center of Zhejiang Province (Hangzhou, China). All the animals were housed in a controlled atmosphere (22 ± 2 °C at 55 ± 5% humidity) with normal light:dark (12:12 h) cycle and free access to food and water. The rats were supplied with water and regular diet and libitum for 7 days of acclimation before starting the study.

Rats were divided into 6 groups (*n* = 8/group), which included: (1) control group (Control); (2) model group (Model); (3) allopurinol group (ALL), in which rats were orally administered with allopurinol (40 mg/kg BW/day); (4) astaxanthin low dose group (ASL-L), in which rats were orally administered with astaxanthin (10 mg/kg BW/day); (5) astaxanthin middle dose group (ASL-M), in which rats were orally administered with astaxanthin (20 mg/kg BW/day) and (6) astaxanthin high dose group (ASL-H), in which rats were orally administered with astaxanthin (40 mg/kg BW/day). The control group received normal drinking water, while the other groups were given drinking water with 10% fructose for six weeks to induce HUA. On day 40, all the rats were singly housed into metabolic cages to collect 24 h urine. At the end of sixth week, the rats were sacrificed and the blood samples were collected for serum preparation. The kidney and liver were isolated and stored at −80 °C for the subsequent experimentations.

### 3.3. Biochemical Analysis

The urine samples were centrifuged at 3000 rpm for 10 min and the supernatants were collected. The whole blood samples were centrifuged at 8000 rpm for 5 min to obtain serum for further analysis. Referring to the method by Zheng et al. [60], the kidney tissues were mixed with normal saline with a ratio of 1:9 on ice, and then centrifuged at 4000 rpm for 10 min to obtain liver homogenate. The bicinchoninic acid (BCA) total protein assay kit was used to quantify the protein concentration in the supernatant of liver homogenate. The levels of BUN, Cr and UA in serum and the enzymatic activities of XOD and ADA were measured using commercial kits (Nanjing Jiancheng Biotechnology Institute, Nanjing, China) according to the manufacturer’s instructions.

### 3.4. RT-PCR Analysis

For the RT-PCR analysis of ADA, XOD, ABCG2, OAT1, OAT3, URAT1 and GLUT9, 0.5 g of liver and kidney tissues were collected. TRIzol reagent was used for the extraction of total RNA according to the manufacturer’s protocol. The cDNA was prepared using HiScript III RT SuperMix from the total RNA. The PCR conditions were set as follows: 50 °C for 2 min, 95 °C for 5 min, then 40 cycles of 95 °C for 15 s and 60 °C for 60 s, 95 °C for 15 s, 60 °C for 15 s. The primer sequences are listed in Table 2. The internal reference and target genes for each sample were subjected to PCR reaction, and each sample was measured in three replicate wells. The experimental data was normalized to β-actin as a housekeeping gene and analyzed by the 2^−ΔΔCt^ method.

### 3.5. Western Blotting

As described by Zheng et al. [60], the protein extraction was carried from kidney sample homogenate at low temperature. The concentration of proteins was detected using BCA protein assay kit. The extracted protein samples were separated using sodium-dodecyl sulfate polyacrylamide gel electrophoresis (SDS-PAGE). After blocking of nonspecific sites, the membrane was probed with primary antibodies (1:1000, anti-URAT1, anti-ABCG2, anti-OAT1, anti-OAT3 and anti-GLUT9, Bioss, Beijing, China) at 4 °C for overnight, which was then followed by incubation with horseradish peroxidase-conjugated secondary antibody (1:2000, Bioss, Beijing, China) at room temperature for 2 h. The images were acquired using Alpha FluorChem FC3 imaging system (ProteinSimple, San Jose, CA, USA) and the expressions of the proteins were analyzed using Image Lab software. β-actin was used as control.

### 3.6. Statistical Analysis

In this study, the data was presented as mean ± SD (standard deviation). The statistical significance between groups was determined by one-way ANOVA, followed by the Tukey–Kramer test (*p* < 0.05) using SPSS version 23 software (Chicago, Illinois, USA).

## 4. Conclusions

The present study investigated the preventive effects of AST on high-fructose-induced hyperuricemia (HUA) in rats. After AST administration, the level of serum UA significantly decreased as compared to the model group. In particular, the high dose of AST restored the serum UA level to normal. From the aspect of UA synthesis, the AST decreased the enzymatic activities of XOD and ADA in serum and the liver by down-regulating the mRNA expression of XOD and ADA in the liver. Meanwhile, the AST also down-regulated the expressions of the mRNA and protein of reabsorption proteins (URAT1 and GLUT9), as well as up-regulated the expressions of the mRNA and protein of secretion proteins (OAT1, OAT3 and ABCG2). Based on these results, it is indicated that the AST decreased the serum UA level by reducing the UA synthesis and promoting the UA excretion. Therefore, the AST could serve as a potential agent for the prevention of HUA.

## Figures and Tables

**Table 1:** Effects of astaxanthin (AST) on 24 h urine volume and total uric acid (UA) excretion.

Group	24 h Urine Volume (mL)	Urine UA (µmol/L)	24 h Total UA Excretion (mg)
Control	22.75 ± 3.40 a	210.36 ± 4.24 c	0.81 ± 0.12 a
Model	90.68 ± 8.28 c	108.90 ± 16.06 a	1.66 ± 0.30 b
Allopurinol	85.00 ± 8.26 bc	123.98 ± 14.33 a	1.78 ± 0.37 b
AST-L	79.16 ± 9.89 bc	116.34 ± 7.34 a	1.54 ± 0.25 b
AST-M	88.25 ± 9.43 bc	132.09 ± 17.06 a	1.86 ± 0.22 b
AST-H	96.50 ± 9.15 c	158.76 ± 13.29 b	2.57 ± 0.29 c

Values were expressed as mean ± SD. Different letters in the same column represent significant differences (*p* < 0.05).

**Table 2:** Primers used in this study.

Products	Forward Primer (5′–>3′)	Reverse Primer (5′–>3′)	bp
ADA	CCTACGAGGGTGCAGTGAAG	TATTGTGTGGTAGCCGTGCC	131
XOD	TGATGGTTCGGTGCTGTTGA	GGGACGGTGTTAGTGCTTGT	128
URAT1	GACCTGCAAGCCCTAGGAAG	CGAAGGATCCCCATCTCACG	194
ABCG2	TAGGTCGGTGTGCGAGTCAG	TAGGATCTATGCCTTTCTAGCTGT	100
OAT1	TTTATGGTGACCCCCACACT	AGCTCTAAATACTTCCAACTGTGA	184
OAT3	CCTGGTGCCATGACCTTCTC	TGGTGGGCTATTCCGAGGAC	120
GLUT9	ATGGACAGCCCATAGATCCG	GTTGTTGACCAGCAGTGTGT	112
β-actin	ACGTCAGGTCATCACTATGG	GGCATAGAGGTCTTTACGGATG	154

## Abbreviations

AST	astaxanthin
UA	uric acid
XOD	xanthine oxidase
ADA	adenosine deaminase
URAT1	urate transporter 1
GLUT9	glucose transporter 9
OAT1	organic anion transporter 1
OAT3	organic anion transporter 3
ABCG2	ATP binding cassette superfamily G 2
Pi	intracellular phosphate
AMPD	AMP deaminase
IMP	inosine monophosphate
PNP	purine nucleoside phosphorylase
BUN	blood urea nitrogen
Cr	creatinine
